# The effect of *Hippophae rhamnoides* L. extract on acrylamideinduced brain injury in rats

**DOI:** 10.1590/ACB361005

**Published:** 2021-11-22

**Authors:** Mehmet Ibrahim Turan, Mehmet Aktaş, Betul Gundogdu, Sevil Karahan Yilmaz, Halis Suleyman

**Affiliations:** 1 MD, Assistant Professor. Department of Pediatric Neurology - Faculty of Medicine - Gaziantep University - Gaziantep, Turkey.; 2 PhD, Assistant Professor. Department of Biochemistry - Faculty of Medicine - Erzincan Binali Yildirim University - Erzincan, Turkey; 3 PhD, Assistant Professor. Department of Pathology - Faculty of Medicine - Ataturk University - Erzurum, Turkey.; 4 PhD, Assistant Professor. Nutrition and Dietetics - Erzincan Binali Yildirim University - Erzincan, Turkey.; 5 PhD, Professor. Department of Pharmacology - Faculty of Medicine - Erzincan Binali Yildirim University - Erzincan, Turkey.

**Keywords:** Hippophae, Acrylamide, Brain Injuries, Toxicity, Rats

## Abstract

**Purpose:**

Reactive oxygen species (ROS), interleukin-1β (IL-1β) and tumor necrosis factor-α (TNF-α) have been shown in the pathogenesis of acrylamide neurotoxicity. *Hippophae rhamnoides* L. extract (HRE) has a cytoprotective effect by stabilizing the production of ROS, IL-1β and TNF-α. The objective of the article was to investigate the effect of HRE on acrylamide-induced brain damage in rats biochemically and histopathologically.

**Methods:**

To the HRE+acrylamide only (ACR) group (n=6) of the animals, HRE was administered orally at a dose of 50 mg / kg into the stomach by gavage. The same volume of solvent (olive oil) was administered orally to the ACR (n=6) and healthy (HG) (n=6) groups. One hour after HRE administration, acrylamide was given orally at a dose of 20 mg/kg to HRE+ACR and ACR groups in the same way. This procedure was repeated once a day for 30 days. At the end of this period, brain tissues extracted from animals killed with 50 mg/kg thiopental anesthesia were examined biochemically and histopathologically.

**Results:**

It has been shown that HRE prevents the increase of malondialdehyde (MDA), myeloperoxidase (MPO), IL-1β and TNF-α with acrylamide and the decrease of total glutathione (tGSH) and glutathione reductase (GSHRd) levels in brain tissue.

**Conclusions:**

HRE may be useful in the treatment of acrylamide-induced neurotoxicity.

## Introduction

Acrylamide (2-propenamid) is an active compound investigated among heat-induced food contaminants^1^. Several studies have reported that various food substances contain differing levels of acrylamide. High levels of acrylamide have been reported in fried and roasted food in particular^2^,^3^. Acrylamide has been documented to exhibit genotoxic, carcinogenic, neurotoxic, reproductive, and developmental effects^4^. Neurotoxicity is an adverse effect that creates damage in the peripheral and central nervous systems in humans and it is dose-dependent^5^. Lipid peroxidation and oxidative stress associated with excessive reactive oxygen species have been implicated in the pathogenesis of acrylamide neurotoxicity^6^. Tabeshpour *et al*.^7^ showed that acrylamide leads to oxidative brain damage by increasing malondialdehyde (MDA) production in brain tissue and reducing endogenous glutathione (GSH) production. Goudarzi *et al*.^8^ showed that acrylamide exhibits neurotoxic effects by increasing the production of both oxidants and proinflammatory cytokines such as interleukin-1ß (IL-1ß) and tumor necrosis factor-a (TNF-a).

*Hippophae rhamnoides* L. extract (HRE), whose protective effect against acrylamide-induced brain damage was investigated in this study, was obtained from the fruit of the *Hippophae rhamnoides* L. (HR) plant (sea buckthorn), a member of the Elaeagnaceae family. HR is traditionally well-known for its pharmacological activities^9^. It contains vitamins A and C, alpha-tocopherol, large quantities of carotenoids, vitamin E, minerals (K, Na, Mg, Ca, Fe, Zn, and Se), monosaccharides, amino acids, flavonoids, fatty acids, glycerol phospholipids, phytosterols, zeaxanthin esters, and polyphenolic compounds^10^. These compounds in the plant exhibit antioxidant, anti-inflammatory, antibacterial, antineoplastic, immunomodulatory, and hepatoprotective effects. Previous studies have shown that HRE possesses potential free radical activity. In addition, HRE has also been reported to be a neuroprotectant against oxidative stress-related neurodegeneration^9^.

HRE has also been reported to exhibit a cytoprotective effect by stabilizing the production of reactive oxygen species (ROS), IL-1ß and TNF-a^11^. This information from the literature indicates that HRE may be beneficial in the treatment of acrylamide-related brain damage. We encountered no previous studies investigating the effect of HRE on acrylamide-induced brain damage.

The purpose of this research was to perform a histopathological and biochemical investigation of the effect on acrylamide-induced brain damage in rats.

## Methods

Animal experiments were performed in accordance with the national guidelines for the use and care of laboratory animals and were approved by the local animal ethics committee of Ataturk University, Erzurum, Turkey (Ethics Committee Number: 75296309-050.01.04-E.2000065782).

Eighteen male albino Wistar rats weighing 285–300 g were procured from the Ataturk University Medical and Experimental Application and Research Center, Erzurum. A seven-day acclimatization period was applied before starting the experiments. The animals were maintained in a 12-h light/darkcycle (lights on 7 a.m. – 7 p.m.) in an air-conditioned constant temperature (22 ± 1°C) colony room, with free access to water and 20% (w/w) protein commercial chow.

### Experimental groups

The experimental animals were divided into healthy (HG), acrylamide only (ACR) and HRE + acrylamide (HRE+ACR) groups.

#### Experimental protocol

Animals in the HRE+ACR group (n = 6) received 50 mg/kg HRE by oral gavage. The ACR (n = 6) and HG (n = 6) groups received the same volume of solvent (olive oil to dilute HRE) by the oral route. One hour after HRE administration, the HRE+ACR and ACR groups received 20 mg/kg acrylamide by the oral route using the same method. These procedures were repeated once daily for 30 days. At the end of that period, the animals were sacrificed under high-dose anesthesia (50 mg/kg thiopental sodium), and the brain tissues were removed and subjected to biochemical and histopathological examination^12^.

### Chemicals

The thiopental sodium used in the experiments was obtained from IE Ulagay (Turkey), HRE from PhytoLab (Russia), and acrylamide from Sigma–Aldrich Chemical Company (United States of America).

### Biochemical procedures

#### Specimen preparation

A pH = 6 potassium phosphate buffer containing 0.5% hexadecyltrimethylammonium bromide was used for MPO assay in brain tissue. For MDA assay, 1.15% potassium chloride solution was made up to 2 mL in pH = 7.5 phosphate buffer for other measurements and homogenized in ice medium. These were than centrifuged at 10,000 rpm for 10 min at +4°C. The supernatant part was used as the analysis specimen.

#### Malondialdehyde analysis

MDA measurement was based on the method described by Ohkawa *et al*.^13^. This method relies on the absorbance of the pink-colored complex formed by thiobarbituric acid (TBA) with MDA at high temperature (95°C) being read spectrophotometrically at a wavelength of 532 nm. The homogenates were centrifuged at 5,000 g for 20 min, and the resulting supernatants were used to calculate the amount of MDA.

#### Myeloperoxidase analysis

In order to perform the MPO assay in brain tissue homogenates, a pH = 6 phosphate buffer containing 0.5% hexadecyltrimethylammonium bromide was prepared. This was then centrifuged for 15 min at 10,000 rpm at +4°C. The resulting supernatant part was used as an analysis specimen. The oxidation reaction with MPO-mediated H_2_O_2_ with 4-amino antipyrine/phenol solution as substrate was used to determine MPO enzyme activity^14^.

#### Total glutathione analysis

The level of GSH in the total homogenate was measured using the method described by Lindsay and Sedlak with some modifications^15^. First, the sample was weighed and homogenized in 2 mL of 50 mmol/L Tris–HCl buffer containing 20 mmol/L ethylenediamine teatraacetic acid (EDTA) and 0.2 mmol/L sucrose at pH 7.5. The homogenate was next immediately precipitated with 0.1 mL of 25% trichloroacetic acid. The precipitate was removed after centrifugation at 4,200 rpm for 40 min at 4°C, and the supernatant was used for GSH level measurement.

#### Glutathione reductase analysis

Glutathione reductase (GSHRd) activity was determined spectrophotometrically by measuring the rate of nicotinamide adenine dinucleotide phosphate (NADPH) oxidation at 340 nm as described by Carlberg *et al*.^16^. Following tissue homogenization, supernatant was used for GSHRd measurement.

#### IL-1ß and TNF-a analysis in tissue

Tissue homogenate IL-1ß and TNF-a concentrations were measured using rat-specific sandwich enzyme-linked immunosorbent assay (ELISA) Rat Interleukin 1ß (Cat. No. YHB0616Ra, Shanghai LZ) and Rat Tumor Necrosis Factor a ELISA (Cat. No. YHB1098Ra, Shanghai LZ) kits. Analyses were performed in line with the manufacturers’ instructions. Briefly, monoclonal antibody specific for rat IL-1ß and TNF-a were coated onto the wells of the microplates. The tissue homogenate, standards, and biotinylated monoclonal antibody specific and streptavidin-HRP were pipetted into these wells and then incubated at 37°C for 60 min. After washing, chromogen reagent A and chromogen reagent B were added. The bound enzyme then reacted with the mixture to produce a color. The mixture was incubated at 37°C for 10 min, after which stop solution was added. The intensity of this colored product was directly proportional to the concentration of rat IL-1ß and TNF-a present in the original specimen. At the end of this procedure, the well plates were read at 450 nm using a microplate reader (Bio-Tek, United States of America). The absorbance of the samples was estimated with formulae using standard graphics.

### Histopathological examination

All tissue samples were first identified in a 10% formaldehyde solution for light microscopic assessment. Following the identification process, the tissue samples were washed under tap water in cassettes for 24 h. The samples were then treated with conventional grade of alcohol (70, 80, 90, and 100%) to remove the water within the tissues. The tissues were then passed through xylol and embedded in paraffin. Four-to-five micron sections were cut from the paraffin blocks and hematoxylin–eosin staining was administered. Their photos were taken following the Olympus DP2-SAL Firmware Program (Olympus® Inc., Tokyo, Japan) assessment. Histopathological assessment was performed by the pathologist blind for the study groups.

### Statistical analysis

The experiment results were expressed as mean ± standard error of the mean (X±SEM). The significance of differences between the groups was determined using the one-way analysis of variance (ANOVA) test, followed by the Tukey test. All statistical procedures were carried out on Statistical Package for Social Sciences 18.0 (Armonk, NY, United States of America) software, and P<0.05 were regarded as significant.

## Results

### Biochemical findings

**Figure 1:**
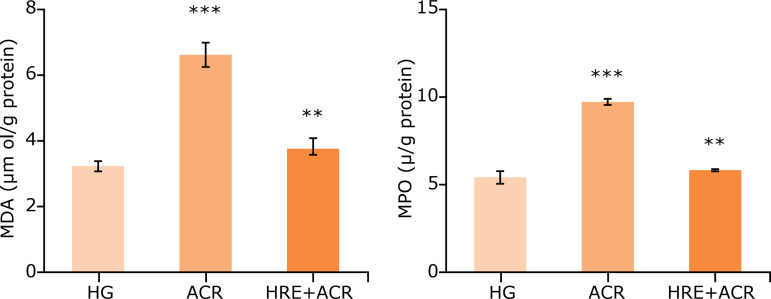
MDA levels and activities of MPO in brain tissue of the study groups.

MDA: malondialdehyde; HG: healthy; ACR: acrylamide only; HRE: *Hippophae rhamnoides* L. extract; MPO: myeloperoxidase; **p < 0.001 according to the ACR group; ***p < 0.001 according to the HG group.

**Figure 2:**
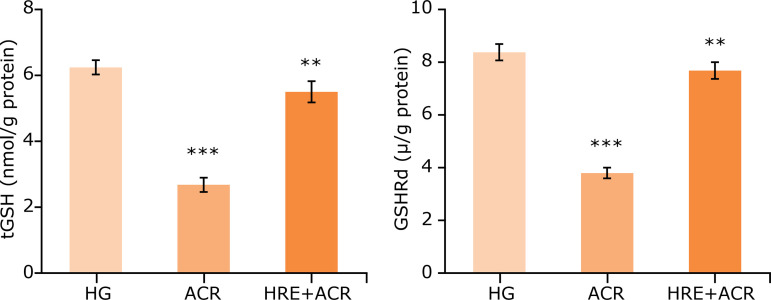
tGSH levels and activities of GSHRd in brain tissue of the study groups.

HG: healthy; ACR: acrylamide only; HRE: *Hippophae rhamnoides* L. extract; tGSH: decrease of total glutathione; GSHRd: glutathione reductase; **p < 0.001 according to the ACR group; ***p < 0.001 according to the HG group.

**Figure 3:**
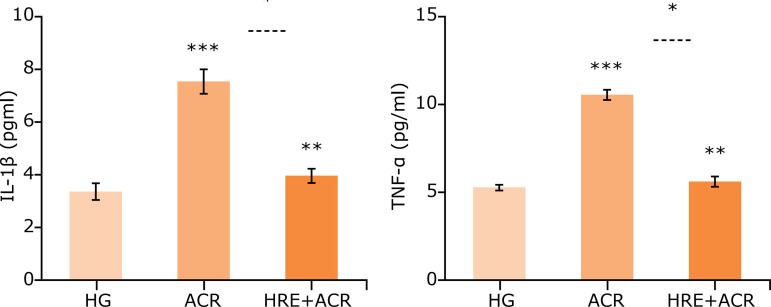
Levels of IL-1ß and TNF-a in brain tissue of the study groups.

HG: healthy; ACR: acrylamide only; HRE: *Hippophae rhamnoides* L. extract; *p < 0.001 according to the ACR group; **p < 0.001 according to the HG group; ***p < 0.001 according to the HG group.

When the ACR group was compared with HG group, a significant increase in MDA levels and MPO activities were noted in brain tissues (p < 0.05). In the HRE+ACR group, the MDA levels and MPO activities were significantly decreased as compared to the ACR group (p < 0.05) (Fig. 1). The level of tGSH, a non-enzymatic endogenous antioxidant, was noted significant decrease in the ACR group in comparison to that in the HG group (p < 0.05). Similarly, GSHRd activities, an enzymatic antioxidant, were significantly lower in the ACR group as compared to that in the HG group (p < 0.05). In the comparison of ACR and HRE+ACR groups, both level of tGSH and GSHRd activities were elevated in favor of the HRE+ACR group (Fig. 2). Additionally, the levels of IL-1ß and TNF-a were significantly higher in ACR groups *vs*. HG groups (p < 0.05). These levels were similar in both HG and HRE+ACR groups (Fig. 3).

### Histopathological findings

**Figure 4:**
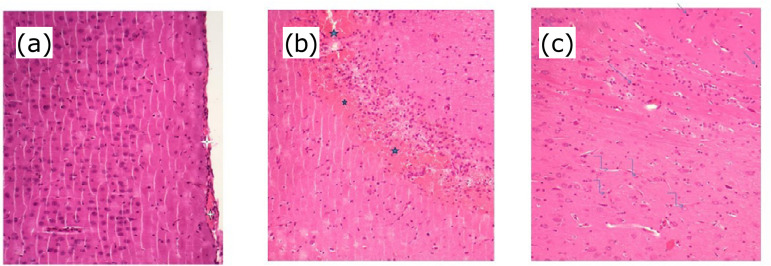
Histopathological appearance of brain tissue the study groups. **(a)** Brain tissue of the HG group: healthy brain tissue with mild congestion (star) (H&E x200). In the section of the cerebral cortex, slight congestion is seen in the tissue structures. **(b)** Brain tissue of the HRE+ACR group: hemorrhage areas (*star*) (H&E x200). **(c)** Brain tissue of the ACR group: inflammatory cell infiltration (*arrow*) and neovascularization (*zigzag arrow*) (H&E x200).

HG: healthy; H&E: hematoxylin–eosin; HRE: *Hippophae rhamnoides* L. extract; ACR: acrylamide only.

As seen in Fig. 4a, no pathological findings were observed in the brain tissue of the HG group, except for mild congestion. However, significant inflammatory cell infiltrate and neovascularization were observed in the brain tissue of the ACR group, which received acrylamide (Fig. 4b). Mild signs of hemorrhage were detected in the brain tissue of the HRE+ACR group treated with HRE (Fig. 4c).

## Discussion

This study investigated the effect of HRE on acrylamide-induced oxidative stress-related neurotoxicity in brain tissue. Various studies have examined the mechanism involved in the central nervous system neurotoxicity caused by acrylamide. In the study of Yousef *et al*.^17^, it was suggested that excessive ROS production is one of the important factors underlying acrylamide neurotoxicity. In addition, it has been reported that oxidative stress is caused by the LPO reaction due to ROS production^18^. As it is known, LPO ends with the production of MDA. However, MDA is cytotoxic and leads to progressive aggravation of cell damage^19^. In recent studies, the success in the treatment of oxidative damage is evaluated by the decrease in MDA level^20^. In the study of Yanji *et al*.^21^, there is information that HRE protects the brain tissue from oxidative damage by reducing the increase in MDA due to toxic substances in the brain. The information obtained from the literature indicates that oxidative stress developed in the brain tissue, as the MDA level was increased in the acrylamide group compared to the healthy and HRE group.

MPO, which was also used in our study to evaluate acrylamide neurotoxicity, is an oxidant-effective enzyme secreted from azurophilic cells of polymorphonuclear leukocyte (PNL) and monocytes^22^. In the literature, there is information that MPO is held responsible in the pathogenesis of many inflammatory diseases, including cardiovascular, kidney, lung, rheumatoid arthritis, skin inflammation and neuronal^23^. The damaging effect of MPO on tissue is enhanced by the formation of hypochlorous acid from hydrogen peroxide and chloride ions^24^. The inhibition of MPO activity by acrylamide by HRE may be due to its inhibition of PNL leakage (infiltration) into the damaged area. It has been documented in previous studies that flavonoids in HRE reduce inflammatory neutrophil and macrophage counts and suppress cell infiltration^25^. The increase in MDA and MPO levels in this study shows that acrylamide-induced neurotoxicity occurs and it is consistent with the literature^26^.

In the literature, the importance of decreasing the intracellular GSH concentration as well as the increase of oxidants in acrylamide-related damage has been expressed^18^. The enzyme that maintains GSH in cells is GSHRd. Therefore, GSH and GSHRd together better protect cells from oxidative damage by ROS^27^. It is known that they provide this cytoprotective effect by converting free radicals and peroxides into harmless products^28^. Our experimental results and literature data show that oxidative stress developed in the brain tissue of the acrylamide group, while oxidative stress was suppressed in the HRE group. Gupta *et al*.^29^ showed that HRE protects brain tissue from toxic damage of ROS products with its antioxidant activity. In addition, it has been reported that HRE prevents the reduction of GSH and other enzymatic and nonenzymatic antioxidants against oxidative damage^30^. It has also been reported that HRE induces the production of GSHRd and other antioxidants^31^.

The role of proinflammatory IL-1ß and TNF-a cytokine increase in the pathogenesis of neurotoxicity in the brain has been stated^32^. The fact that IL-1ß and TNF-a levels were elevated in the acrylamide group in our study is consistent with studies showing inflammation in the pathogenesis of neurotoxicity^33^. The low levels of proinflammatory cytokines in the group receiving HRE are in line with the literature reporting its anti-inflammatory effect^34^. Decreased IL-1ß and TNF-a levels due to suppression of nuclear factor kappa B (NF-kB) expression are held responsible for the anti-inflammatory mechanism of action of HRE^35^. HRE fruits have been reported to contain carotenes, fatty acids, and many water- and fat-soluble vitamins that have antioxidant, anti-inflammatory, and antiulcer IL-1ß and TNF-a antagonist features^36^.

Histopathologically, neovascularization and inflammatory cell infiltrate were observed in the acrylamide group in which oxidant and proinflammatory cytokine levels were measured high. This shows that our biochemical test results are consistent with the histopathological findings. A number of inflammatory cells, including neutrophils, eosinophils, mast cells, macrophages, is known to be involved in inducing angiogenesis^37^. In addition, it is stated that the important link between inflammation and neovascularization is oxidative stress^38^. No information was found in the literature to support the protective effect of HRE on acrylamide-induced neurotoxicity in brain tissue. However, there is information that HRE improves histopathological changes in the cerebral cortex in arsenic and lead-induced neurotoxicity in mice^39^.

## Conclusion

HRE prevented the increase in acrylamide-related oxidant and cytokines and the decrease in antioxidants. In addition, HRE has been shown histopathologically to prevent acrylamide-related brain oxidative and inflammatory damage. Based on our experimental results, we think that HRE may be an effective product in preventing acrylamide-related neurotoxicity. More detailed studies are needed in the future to clarify the protective effect mechanism of HRE against acrylamide-related brain damage.
